# One‐Stage Bilateral Pulmonary Nodule Resection via Unilateral Thoracic Cavity Access: A Single‐Center Experience of 12 Cases

**DOI:** 10.1111/1759-7714.70053

**Published:** 2025-03-24

**Authors:** Zhen Wang, Zhaolei You, Yingjian Song, Hua Ji, Guodong Jiang, Xiaokun Bu, Jingyu Zhang, Tengfei Yi, Jian Fang, Xiaofeng Yu

**Affiliations:** ^1^ Department of Thoracic Surgery Yantai Yuhuangding Hospital, Qingdao University Yantai People's Republic of China; ^2^ Department of Cardiothoracic Surgery The People's Hospital of Zhaoyuan City Yantai People's Republic of China; ^3^ Department of Cardiothoracic Surgery Yantai Yeda Hospital Yantai People's Republic of China

**Keywords:** bilateral pulmonary nodules, one‐stage resection, unilateral transcostal incision

## Abstract

**Background:**

Surgical intervention remains the primary therapeutic modality for managing multiple pulmonary nodules. However, in cases with bilateral pulmonary nodules, one‐stage bilateral resection is discouraged due to tumor aggressiveness and surgical invasiveness. In light of this, we investigated an innovative approach, termed one‐stage bilateral pulmonary nodule resection via unilateral thoracic cavity access.

**Methods:**

From July 2022 to September 2024, a cohort of 12 patients with bilateral pulmonary nodules were enrolled in this study. This technique involves initial unilateral transcostal incision for segmental or lobectomy of a nodule on one side, followed by bilateral mediastinal pleura incision through the anterior mediastinum, facilitating subsequent wedge resection of the contralateral nodule. Clinical and pathological data, along with perioperative imaging findings and follow‐up information, were systematically collected and subjected to a comprehensive retrospective analysis.

**Results:**

A total of 25 nodules were resected from 12 patients. Regarding surgical approaches, nine patients underwent right thoracic incision, while three patients underwent left thoracic incision. Intraoperatively, seven patients received bilateral wedge resections, whereas five patients underwent segmentectomy on one side combined with wedge resection on the contralateral side. R0 resection of the contralateral nodules was successfully achieved during the procedures. The average distance between the surgical margin and the contralateral nodules was 12.5 mm, ranging from 5 mm to 25 mm. Of the 12 patients, one (Patient 6) was lost to follow‐up, while the remaining 11 patients underwent postoperative chest CT examinations. The median follow‐up duration for these 11 patients was 105 days (range: 36–857 days). No evidence of bilateral pleural effusion or tumor recurrence was detected on follow‐up chest CT scans.

**Conclusions:**

This study offers the potential to concurrently address bilateral pulmonary nodules, thereby sparing patients from the need for a subsequent hospitalization for surgical intervention.

## Graphical abstract

The standard surgical procedure for patients with synchronous bilateral pulmonary nodules remains contentious. An innovative procedure, termed one‐stage bilateral pulmonary nodule resection via unilateral thoracic cavity access, was performed in these patients.

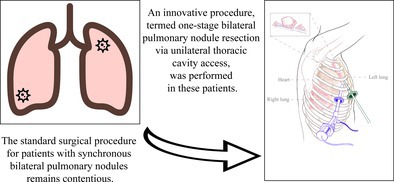

## 1 Introduction

Lung cancer remains the malignant tumor with the highest morbidity and mortality worldwide [1]. Low dose computed tomography (LDCT) screening has emerged as a promising approach to reduce lung cancer‐related deaths among high‐risk populations [2]. With the popularization of LDCT screening, more and more pulmonary nodules are being detected, including cases with bilateral pulmonary nodules [3]. Surgical intervention remains the primary treatment for high‐risk nodules, yet the optimal approach for patients with synchronous bilateral pulmonary nodules remains contentious.

For these patients, the predominant treatment across most medical centers involves staged surgical interventions. However, an emerging trend within certain medical centers involves the exploration of simultaneous resection methodologies for bilateral pulmonary nodules, utilizing both bilateral thoracic approaches and the subxiphoid approach [4, 5, 6, 7].

In light of this, we investigated an innovative approach, termed one‐stage bilateral pulmonary nodule resection via unilateral thoracic cavity access. This study was approved by the Ethics Committee of Yantai Yuhuangding Hospital (approval number: 2024‐210).

## 2 Methods

### 2.1 Patients

The inclusion criteria for selecting patients in this study are as follows: (1) Simultaneous preoperative diagnosis of bilateral pulmonary nodules, both meeting surgical indications based on imaging evaluations; (2) Necessity for sublobectomy or lobectomy on one side, with the contralateral pulmonary nodule located in the middle or anterior regions of the lung field, allowing R0 resection via wedge resection; (3) Normal cardiopulmonary function with no contraindications for surgery. Exclusion criteria include: (1) Abnormal cardiopulmonary function precluding the feasibility of simultaneous bilateral lung surgery; (2) Refusal to undergo preoperative CT‐guided puncture localization of pulmonary nodules. Informed consent was obtained from all patients.

Preoperative evaluation protocol for patients includes chest CT and 3D reconstruction of nodules, brain magnetic resonance imaging (MRI) or brain CT, abdominal and adrenal gland ultrasound, electrocardiogram, cardiac color doppler ultrasound, and pulmonary function test. For patients with solid tumors larger than 2 cm in diameter, a whole‐body bone scan is conducted. Additionally, patients undergo multidisciplinary consultations involving specialists in imaging, radiotherapy, and medical oncology to optimize surgical planning. All patients in this study were staged cN0M0 after evaluation.

### 2.2 Surgical Procedure

The preoperative evaluation determines the appropriate surgical approach for each nodule. For nodules on one side, sublobectomy or lobectomy is performed, while nodules on the contralateral side are addressed through wedge resection. Prior to surgery, a CT‐guided localization technique is employed to accurately position the contralateral nodule intended for wedge resection.

All patients underwent general anesthesia and single‐lumen endotracheal intubation and were positioned in the lateral decubitus position. Unilateral lung ventilation was achieved using a bronchial occluder. The primary surgical incision, approximately 3–4 cm in length, was made in the fourth or fifth intercostal space along the anterior axillary line, with an incision protector in place. Additionally, a 1 cm observation port was created in the seventh intercostal space along the mid‐axillary line.

**FIGURE 1:**
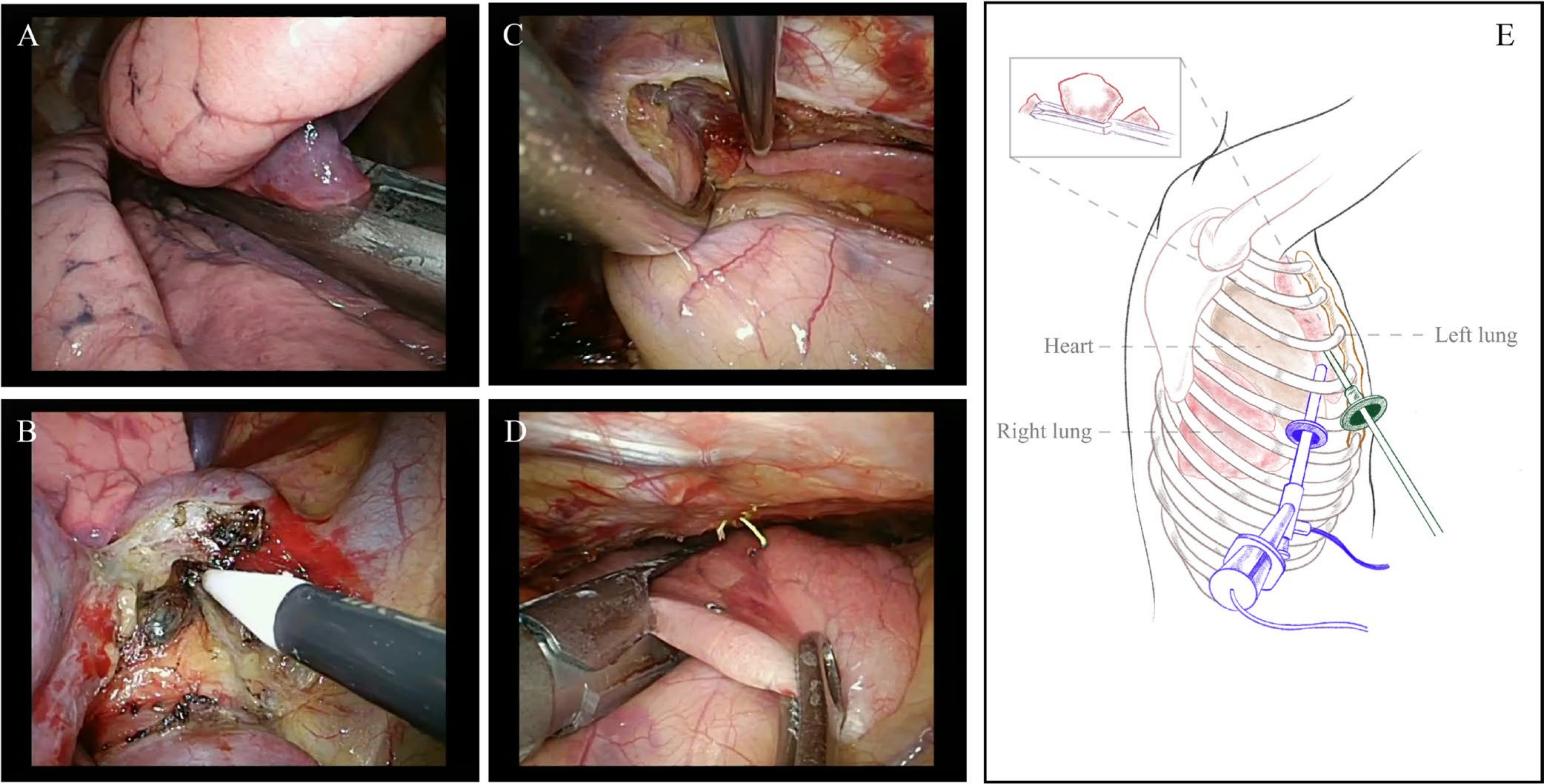
(A) Perform a wedge resection to remove the nodule in the right lung. (B) Hilar lymph nodes in the right thorax were biopsied. (C) The mediastinal pleura was incised to expose the left lung. (D) A wedge resection of the left lung nodule was conducted according to its preoperative localization. (E) A schematic diagram illustrates the surgical procedure of one‐stage bilateral pulmonary nodule resection via unilateral thoracic cavity access.

Initially, unilateral sublobectomy or lobectomy is performed. Systematic lymph node dissection is conducted if frozen pathology suggests invasive lung cancer. Subsequently, the bilateral mediastinal pleurae are incised in the anterior mediastinum to expose the contralateral thoracic cavity. Thoracoscopy is then utilized to visualize the contralateral thorax through the anterior mediastinal space and accurately locate the contralateral pulmonary nodule with the help of the puncture needle. Upon confirmation of the nodule's location, long oval forceps are employed to grasp and lift lung tissue, and the diseased tissue is excised using a curved endoscopic cutting stapler. Frozen pathology examines the distance between the pulmonary nodule and the resection margin to ensure R0 resection. The surgical process and schematic diagram are shown in Figure 1. Postoperatively, a 22# drainage tube is inserted through the observation port, while no drainage tube is placed in the contralateral chest.

The patients received nonsteroidal drugs for analgesia after surgery. The intensity of postoperative pain was assessed using the Visual Analog Scale (VAS). On the first day after surgery, bedside anteroposterior chest X‐rays are performed to assess for pneumothorax or pleural effusion in both chests. Removal of the chest drainage tube is considered when the daily drainage volume is below 200 mL and there is no evidence of air leakage in the chest drainage system. During postoperative follow‐up, patients will undergo chest CT scans to evaluate for issues such as tumor recurrence and pleural effusion.

The clinical profiles, pathological findings, and postoperative results of surgical procedures were then collected and studied.

## 3 Results

From July 2022 to September 2024, a cohort of 12 patients underwent one‐stage bilateral pulmonary nodule resection via unilateral thoracic cavity access in our department. The cohort comprised three males and eight females, with a mean age of 53.8 years (range: 35–69 years). The patients' average body mass index (BMI) was 26.4 kg/m^2^ (range: 18.1–34.8 kg/m^2^). Among them, one patient (patient 5) had previously undergone right upper lobectomy and mediastinal lymph node dissection for lung cancer, while the remaining patients had no history of malignant tumors. All patients exhibited normal pulmonary function in preoperative assessments. The clinical characteristics of all patients are shown in Table 1.

**TABLE 1:** Clinical characteristics and imaging features of 12 patients.

No.	Age (years)	Gender	BMI (kg/m^2^)	Tumor marker	Nodule number (R/L)	Nodule location (R/L)	Imaging features (R/L)	Maximum diameter (mm) (R/L)
Patient 1	60	Female	26.6	AFP elevated	1/1	RU/LU	mGGN/pGGN	11/13
Patient 2	61	Male	27.4	NSE elevated	2/1	RU + RU/LU	mGGN + mGGN/pGGN	10 + 9/10
Patient 3	54	Female	26.7	Normal	1/1	RU/LU	mGGN/pGGN	8/4
Patient 4	57	Female	20.6	Normal	1/1	RU/LL	mGGN/mGGN	5/6
Patient 5	64	Male	20.9	SCCA elevated	1/1	RL/LU	SN/pGGN	50/11
Patient 6	59	Female	34.8	Normal	1/1	RL/LU	mGGN/mGGN	22/12
Patient 7	42	Female	34.3	NSE elevated	1/1	RL/LL	mGGN/pGGN	16/5.5
Patient 8	69	Female	29.1	Cyfra 21‐1 and NSE elevated	1/1	RM/LU	mGGN/pGGN	13/7
Patient 9	37	Female	28.2	Normal	1/1	RU/LU	pGGN/pGGN	8/4
Patient 10	59	Female	24.4	Normal	1/1	RL/LU	mGGN/mGGN	5/5.5
Patient 11	35	Male	25.2	Normal	1/1	RL/LL	pGGN/mGGN	5/13
Patient 12	49	Female	18.1	Normal	1/1	RU/LU	pGGN/pGGN	8/11

Abbreviations: AFP, alpha‐fetoprotein; BMI, body mass index; L, left; LL, left lower lobe; LU, left upper lobe; mGGN, mixed ground‐glass nodule; NSE, neuron specific enolase; pGGN, pure ground‐glass nodule; R, right; RL, right lower lobe; RM, right middle lobe; RU, right upper lobe; SCCA, squamous cell carcinoma antigen; SN, solid nodule.

**FIGURE 2:**
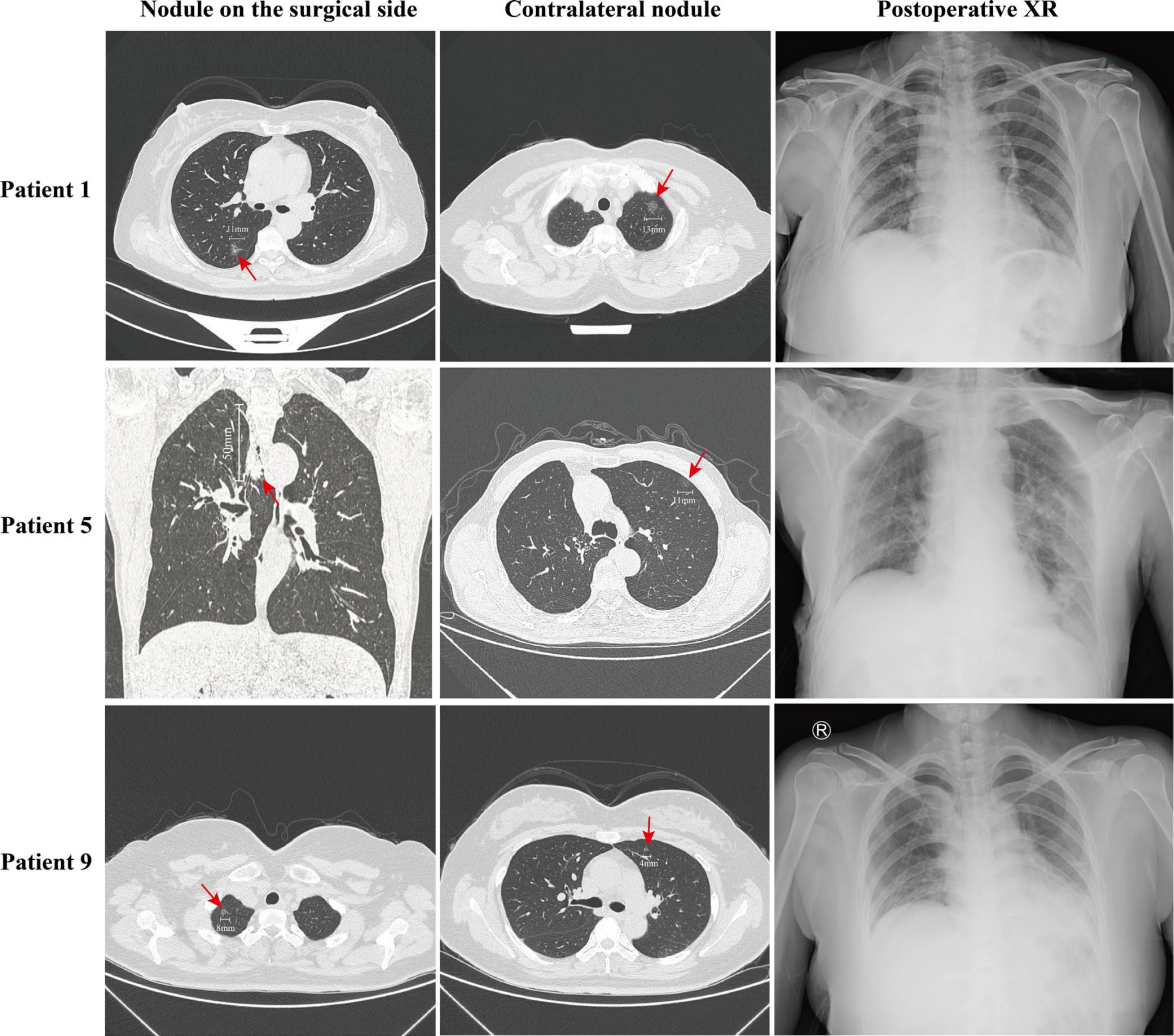
Typical imaging pictures of bilateral pulmonary nodules and postoperative anteroposterior chest radiographs of patient 1, patient 5, and patient 9. (XR, X‐radiography).

A total of 25 nodules were excised from these 12 patients. Among these, 11 patients had one nodule in each lung, while one patient presented with two nodules in the right lung and one nodule in the left. Preoperative chest CT scans identified 13 mixed ground‐glass nodules (mGGNs), 11 pure ground‐glass nodules (pGGNs), and one solid nodule (SN) among the total 25 lung nodules. Typical imaging pictures are shown in Figure 2.

Regarding the surgical approach, nine patients underwent right thoracic incision, and three patients underwent left thoracic incision. Intraoperatively, seven patients received wedge resection of both lungs, while five patients underwent segmentectomy on one side and wedge resection on the other. Among these cases, one patient (patient 5) was preoperatively staged as cT2bN0M0 for a nodule in the right lower lobe. However, considering the prior resection of the right upper lobe and the necessity to preserve lung function, only a wedge resection of the right lower lobe was performed.

Among the 13 mGGNs, four were diagnosed as invasive adenocarcinomas, six as microinvasive adenocarcinomas, and three as benign lesions. Of the 11 pGGNs, five were diagnosed as microinvasive adenocarcinomas, five as adenocarcinoma in situ, and one as a benign lesion. Additionally, the sole SN was diagnosed as invasive adenocarcinoma. The pathological diagnoses and details of the surgical margins are provided in Table 2.

**TABLE 2:** Postoperative outcomes and pathological results of 12 patients.

Patient	Operation side	Surgery options (R/L)	Operation time (min)	Postoperative VAS	Postoperative XR	Pathology (R/L)	Margin (mm) (R/L)	Pathological stage (R/L)	Follow‐up time (days)
Patient 1	R	S/W	185	4	Normal	ADC (60% LPA + 40% APA)/MIA	50/5	pT1bN0M0 IA2/pT1aN0M0 IA1	857
Patient 2	R	W + W/W	110	7	Normal	ADC (60% APA + 40% LPA) + MIA/AIS	12 + 10/5	pT1aN0M0 IA1 + pT1aN0M0 IA1/pTisN0M0	36
Patient 3	R	S/W	155	3	Normal	MIA/MIA	25/10	pT1aN0M0 IA1/pT1aN0M0 IA1	105
Patient 4	R	W/W	85	3	Normal	MIA/MIA	18/15	pT1aN0M0 IA1/pT1aN0M0 IA1	316
Patient 5	R	W/W	135	5	Normal	ADC (80% APA + 20% SPA)/AIS	2/13	pT3N0M0 IIB/pTisN0M0	225
Patient 6	R	S/W	120	3	Normal	ADC (70% APA + 20% LPA + 10% MPA)/ADC (60% APA + 40% LPA)	40/18	pT1cN0M0 IA2/pT1bN0M0 IA3	Loss
Patient 7	R	W/W	105	4	Normal	Benign/MIA	15/10	‐/pT1aN0M0 IA1	169
Patient 8	L	W/W	110	5	Normal	Benign/AIS	10/8	‐/pTisN0M0	36
Patient 9	R	W/W	90	3	Normal	MIA/AIS	10/14	pT1aN0M0 IA1/pTisN0M0	120
Patient 10	R	W/W	80	6	Normal	BA/MIA	40/15	‐/pT1aN0M0 IA1	36
Patient 11	L	W/S	115	5	Normal	AIS/MIA	10/20	pTisN0M0/pT1bN0M0 IA2	43
Patient 12	L	W/S	115	4	Normal	Benign/MIA	25/25	‐/pT1aN0M0 IA1	94

Abbreviations: ADC, adenocarcinoma; AIS, adenocarcinoma in situ; APA, acinar pattern of adenocarcinoma; BA, bronchiolar adenoma; L, left; LPA, lepidic predominant adenocarcinoma; MIA, minimally invasive adenocarcinoma; MPA, micropalillary pattern of adenocarcinoma; R, right; S, segmentectomy; SPA, solid pattern of adenocarcinoma; W, wedge resection; XR, X‐radiography.

All surgeries were completed successfully, with an average duration of 117.1 min (range: 80–185 min). Notably, there were no occurrences of intraoperative bleeding, conversions to thoracotomy, or other significant complications. During the operation, R0 resection of the contralateral nodules was achieved. The mean distance between the surgical margin and the nodules was 12.5 mm, with a range from 5 to 25 mm.

Postoperative anteroposterior chest radiographs on the first day revealed no apparent pneumothorax or pleural effusion in any patient. Postoperative pain was managed with nonsteroidal analgesics, with the average pain score reported as 4.25, ranging from 3 to 7.

Among the 12 patients, one (patient 6) was lost to follow‐up, while the remaining 11 patients underwent postoperative chest CT examinations. The median follow‐up period for the 11 patients was 105 days (range: 36–857 days), and no signs of bilateral pleural effusion or tumor recurrence were detected on the chest CT scans.

## 4 Discussion

Lung cancer remains the leading cause of cancer‐related mortality globally [1]. Thus, the early detection and treatment of lung cancer are critical in reducing the associated deaths. Prior research has demonstrated that LDCT screening can significantly lower the mortality rate among high‐risk populations [2]. Concurrently, the widespread adoption of LDCT screening has led to a higher detection rate of pulmonary nodules, with some patients exhibiting multiple nodules during examinations [3, 8].

Previous studies have demonstrated that over 15% of the population exhibits multiple pulmonary nodules, with this incidence rising annually [9, 10]. These nodules can be generally classified into benign and malignant types. Malignant nodules, in particular, may signify the presence of multiple primary lung cancers [11, 12].

In the diagnosis and treatment of multiple pulmonary nodules, particularly multiple primary lung cancers, surgical intervention remains the preferred approach [13, 14, 15]. The American Joint Committee on Cancer (AJCC) guideline advocates for sublobectomy for all multiple nodules suspected to be malignant when technically feasible, while discouraging pneumonectomy [10, 16]. Throughout the surgical process, it is crucial to adhere strictly to oncological principles and to prioritize the preservation of lung function as much as possible.

Previous studies have demonstrated that radical resection of multiple primary lung cancers may be the optimal treatment option, providing a favorable long‐term prognosis [17, 18]. Currently, most medical centers adopt a staged surgical treatment model, wherein the patient initially undergoes resection of unilateral lung nodules, followed by a subsequent surgery to remove the contralateral nodules after a certain interval [19]. This approach aims to mitigate perioperative risks but results in two separate surgical interventions, thereby increasing patient trauma and overall treatment costs [20].

In an effort to optimize the treatment model for patients with bilateral multiple primary lung cancers, many centers have adopted simultaneous surgical strategies. Some centers employ video‐assisted thoracoscopic surgery (VATS) to perform bilateral lung nodule resection concurrently, and its perioperative safety has been validated [5, 7]. This approach allows for complex lung segment resections in both chest cavities, but bilateral chest wall incisions result in increased trauma to the patient. Moreover, this surgical procedure is associated with prolonged operation times, with previous reports indicating an average duration of 220–260 min [5, 21]. Alternatively, other centers have explored a subxiphoid incision for bilateral lung nodule resections [4, 6]. This approach reduces patient trauma but limits the surgeon to performing only simple wedge resections in both chest cavities.

Therefore, we explored a one‐stage bilateral pulmonary nodule resection via unilateral thoracic cavity access. Initially, nodule resection and lymph node biopsy or dissection were performed within one thorax. Subsequently, the bilateral mediastinal pleura was incised, enabling a simple wedge resection of the contralateral nodule through the anterior mediastinal space.

In comparison to the subxiphoid approach, our surgical technique facilitates complex lung segmentectomy and lymphadenectomy within one thorax, thereby achieving radical treatment of the primary lesion. Unlike simultaneous bilateral thoracoscopic procedures involving dual incisions, our method minimizes patient trauma and, to a degree, reduces the overall surgical time. Additionally, we achieved R0 resection for all contralateral nodules during the procedure, with an average distance of 12.5 mm (range: 5–25 mm) from the resection margin to the nodule.

A comprehensive preoperative evaluation of the patient is essential. Based on our current experience, when the contralateral nodule is situated in the middle or anterior regions of the lung field, especially within the anterior third, it is generally feasible to sufficiently expose the nodule during the procedure. Additionally, as intraoperative palpation is not available for nodule localization, preoperative CT‐guided localization is essential. The precision of this localization is crucial for ensuring an accurate R0 resection of the contralateral nodule. In this study, the patients' average BMI reached 26.4 kg/m^2^, with two patients having a BMI greater than 30 kg/m^2^. Based on our experience, removing part of the anterior mediastinal fat tissue during surgery can adequately expose the contralateral pleural cavity in obese patients, allowing for successful surgical procedures.

Consequently, for patients with bilateral pulmonary nodules, particularly when one nodule is situated in the central or anterior portion of the lung field, and following a comprehensive preoperative evaluation, thoracoscopic resection of both nodules via unilateral thoracic cavity access may be regarded as a feasible surgical approach.

It is crucial to note that this study is a retrospective cohort analysis with a relatively small sample size and lacks a control group, which may introduce selection bias. Although CT scans were conducted during the postoperative follow‐up, one patient was lost to follow‐up, and the follow‐up period was relatively short. Therefore, further prospective studies with larger sample sizes and extended follow‐up periods are necessary.

## 5 Author Contributions

Xiaofeng Yu was the leading surgeon and provided a critical revision of the article. Zhen Wang was the main author of this article. Jian Fang and Zhaolei You contributed substantially to the planning of the surgery. Yingjian Song, Hua Ji, Guodong Jiang, Xiaokun Bu, Jingyu Zhang and Tengfei Yi provided significant help in the pre‐ and postoperative therapy.

## Ethics Statement

The authors are accountable for all aspects of the work in ensuring that questions related to the accuracy or integrity of any part of the work are appropriately investigated and resolved. The study was conducted in accordance with the Declaration of Helsinki (as revised in 2013). The study was approved by ethics committee of Yantai Yuhuangding hospital on March 25, 2024 (approval No. 2024‐183).

## Consent

The patient has signed an informed consent and agreed to publish all images, clinical data, and other data included in the manuscript.

## Conflicts of Interest

The authors declare no conflicts of interest.

## Supporting information


Data S1.

Video S1.

## Data Availability

The original contributions presented in the study are included in the article/Supporting Information. Further inquiries can be directed to the corresponding authors.
