# Supplementary material for: One‐Stage Bilateral Pulmonary Nodule Resection via Unilateral Thoracic Cavity Access: A Single‐Center Experience of 12 Cases
Source: Thorac Cancer. 2025 Mar 24;16(6):e70053. doi: 10.1111/1759-7714.70053 (PMC11933441; doi:10.1111/1759-7714.70053)
Supplement: Supplementary file 1 — Data S1. [file TCA-16-e70053-s002.docx]

Surgical procedure video of patient 11.
